# Constraint-induced intervention as an emergent phenomenon from synaptic competition in biological systems

**DOI:** 10.1007/s10827-021-00782-9

**Published:** 2021-04-06

**Authors:** Won J. Sohn, Terence D. Sanger

**Affiliations:** 1 Department of Neurology, University of California at Irvine, 200 S. Manchester Ave, Orange, CA 92868 USA; 2 Department of Biomedical Engineering, University of Southern California, 1042 Downey Way, Los Angeles, CA 90089 USA; 3 Department of Biokinesiology, University of Southern California, 1042 Downey Way, Los Angeles, CA 90089 USA; 4 Department of Neurology, University of Southern California, 1042 Downey Way, Los Angeles, CA 90089 USA

**Keywords:** Spike-timing-dependent plasticity (STDP), BCM theory, Constraint-induced movement therapy, Hemiplegic cerebral palsy, Synaptic competition

## Abstract

The principle of constraint-induced therapy is widely practiced in rehabilitation. In hemiplegic cerebral palsy (CP) with impaired contralateral corticospinal projection due to unilateral injury, function improves after imposing a temporary constraint on limbs from the less affected hemisphere. This type of partially-reversible impairment in motor control by early brain injury bears a resemblance to the experience-dependent plastic acquisition and modification of neuronal response selectivity in the visual cortex. Previously, such mechanism was modeled within the framework of BCM (Bienenstock-Cooper-Munro) theory, a rate-based synaptic modification theory. Here, we demonstrate a minimally complex yet sufficient neural network model which provides a fundamental explanation for inter-hemispheric competition using a simplified spike-based model of information transmission and plasticity. We emulate the restoration of function in hemiplegic CP by simulating the competition between cells of the ipsilateral and contralateral corticospinal tracts. We use a high-speed hardware neural simulation to provide realistic numbers of spikes and realistic magnitudes of synaptic modification. We demonstrate that the phenomenon of constraint-induced partial reversal of hemiplegia can be modeled by simplified neural descending tracts with 2 layers of spiking neurons and synapses with spike-timing-dependent plasticity (STDP). We further demonstrate that persistent hemiplegia following unilateral cortical inactivation or deprivation is predicted by the STDP-based model but is inconsistent with BCM model. Although our model is a highly simplified and limited representation of the corticospinal system, it offers an explanation of how constraint as an intervention can help the system to escape from a suboptimal solution. This is a display of an emergent phenomenon from the synaptic competition.

**Supplementary Information:**

The online version contains supplementary material available at 10.1007/s10827-021-00782-9.

## Introduction

Constraint-induced movement therapy (CIMT) improves limb use in patients after unilateral cerebrovascular accidents (CVA) (Taub et al., 1999), and other asymmetric injuries to sensory or motor areas of the central nervous system (CNS) (Miltner et al., 1999). The principle of the technique is to constrain the use of the less-impaired upper extremity while intensive and repetitive training of motor activities in the more impaired extremity are promoted for up to 6 h per day during 2–4 weeks (Brady & Garcia, 2009; Taub et al., 1999, 2007). The resulting improvement of function years or decades following stroke has led to a complete rethinking of the role of plasticity in rehabilitation of CNS injury (Wolf et al., 2006). Although the causal link between the rehabilitative practice and the clinical outcome has been supported by clinical investigations, there is a considerable gap between this emerging clinical practice and a computational understanding of the neural mechanism underlying the therapeutic method. We strive to overcome this gap by leveraging a neuromorphic high-speed hardware emulation platform to emulate the neural underpinnings of constraint-induced therapy.

Conceptually similar interventional strategies that increase the activity in injured areas relative to uninjured areas in an animal model of hemiplegic cerebral palsy (CP) have been shown to contribute to significant corticospinal tract (CST) repair and motor recovery (Friel et al., 2014). In a feline model of CP, Martin et al*.* found that with hemiplegic CP, the impaired contralateral CST is at disadvantage early in development, compared with the ipsilateral CST that is undamaged, in forming a strong synaptic connection in the spinal cord (Martin et al., 2011). It was proposed that activity-dependent synaptic competition is a key mechanism in perpetuation of deficits, as well as in repairing the damaged CST and restoring skilled motor function. The experiment is significant in understanding the development and treatment of postnatal hemiplegic cerebral palsy. Note that this mechanism may apply primarily to older children and adults; in animal studies and in children with hemiplegic CP prior to the first 24 months, the plasticity may be due to ipsilateral CST innervation of spinal neurons including motoneurons (Eyre, 2007; Eyre et al., 2007), whereas later onset stroke in humans may engage recovery of CST and areas nearby to the normal motor output areas contralateral to the weakness (Hallett et al., 1998).

Martin et al. showed that temporarily imposed constraint in the less affected hemisphere and active stimulation in the more affected hemisphere harness activity-dependent plasticity to restore the diminished connection strength (Friel & Martin, 2007; Martin, 2005; Martin et al., 2011). When activity in one motor cortex is temporarily blocked pharmacologically during an early critical developmental period, CST axons withdraw their projections (Friel & Martin, 2007). When voluntary use of one limb is temporarily prevented during a critical developmental period, similar effects on the development of contralateral CST projections are observed (Martin et al., 2004).

A key element in both cases is that a temporary intervention (pharmacological blockade or reduced behavioral use) leads to persistent deficits that are manifested by structural changes in connectivity downstream from the causative abnormality. In other words, a change in behavior or cortical stroke can lead to persistent plastic changes in the otherwise unaffected spinal cord. The effectiveness of CIMT is therefore based upon two hypotheses: (1) some component of the persistent deficit is due to persistent yet reversible effects of plasticity, and (2) the component of the deficit due to plasticity can be ameliorated by interventions that changes the ratio of CNS activity between the injured and uninjured hemispheres.

This phenomenon is similar to what is seen in amblyopia caused by asymmetric and temporary visual disruption during early development (Attebo et al., 1998). Effective treatment for amblyopia includes patching the more functional eye thus forcing the child to use the weaker eye (Holmes et al., 2003). In animal studies, the effect of monocular deprivation is reversible only if the treatment is applied during a critical period because the deprivation otherwise causes permanent changes in visual cortex (Movshon, 1976; Movshon & Van Sluyters, 1981) although it is reported that in human the period during which the recovery from amblyopia is obtained can be extended to the teenaged years or even into adult years (Daw, 1998). A common hypothesis is that patching of an unaffected eye provides a competitive advantage to signals from the amblyopic eye, allowing them to overcome and reverse the abnormal ocular dominance perpetuated by plasticity due to early asymmetric visual deprivation. It is not known whether the effect of patching in amblyopia and CIMT share similar mechanisms, but the phenomenon of persistent asymmetric deficits that are at least partially reversible through behavioral interventions suggests that there may be at least a functional similarity.

Previously, the treatment mechanism for amblyopia has been understood within the framework of BCM (Bienenstock-Cooper-Munro) theory (Bienenstock et al., 1982), a rate-based synaptic modification theory. The theory provides a first-order approximation to plasticity and long-term depression (LTD) in visual cortex (Kirkwood et al., 1993). Although the prediction from the theory is largely consistent with the ocular dominance changes in amblyopia, it remains to this day that the high-level phenomenological model can be supplemented by models that retain the characteristics of biological mechanism underlying synaptic plasticity.

Here, we attempt to provide a framework that accounts for an inter-hemispheric synaptic competition based on spike-based plasticity theory. We emulate the development and restoration of abnormal motor connectivity in hemiplegic cerebral palsy, using the recently available technology of field-programmable-gate-arrays (FPGAs). With FPGA, the neuronal activity can be accelerated to 190 × real-time, therefore we are able to observe weeks of neural development within hours. The BCM-like rate-based model requires an explicit homeostatic plasticity mechanism, whereas spike-timing-dependent plasticity (STDP) in this study shows that no additional mechanism is required. It is advantageous to use spike-based emulation because the behavior of spiking algorithm could be different from the rate-base algorithm. This will be particularly true in the context of injury, which affects individual cells and is more difficult to emulate accurately in rate-based models. Our purpose is to support a mechanistic hypothesis to explain CIMT by showing that STDP is sufficient to predict: (1) persistence of plasticity-mediated deficits following temporary injury, (2) reversal of plasticity-mediated components of deficit via constraint therapy, and (3) persistence of improved function following termination of constraint therapy. Although our model is a highly simplified and limited representation of the motor system, it allows us to understand how the principle of constraint-induced therapy can be explained in terms of synaptic competition through activity-dependent plasticity. By finding a minimally complex, yet sufficient condition to demonstrate the effect of constraint-induced intervention could shed light on the effective practices of clinical therapies.

## Materials and methods

We customized a two-layer neuronal network from our recently developed neuromorphic hardware (Niu et al., 2012, 2014; Sohn et al., 2015, 2016) (Jalaleddini et al., 2017; Niu et al., 2017). The hardware emulation of the spiking neurons and the associated neural structure are constructed using field programmable gate arrays (FPGA, Xilinx Spartan-6), a programmable version of VLSI electronic chips. The FPGA communicates with a data-logging computer through a high-speed USB channel, OpalKelly development kit and interface software (XEM6010, OpalKelly Inc.). The development kit includes 128-MiB DDR2 SDRAM, 32-Mib serial flash, 4,824 Kib of block RAM, max central clock frequency of 200 MHz, and supports full FrontPanel virtual control through the computer. Xilinx design environment (Xilinx Vivado Design Suite) is used as a design tool. Our arithmetic implementations are compatible with IEEE-754 standard. The source code in Verilog hardware descriptive language (Verilog-2001, IEEE Standard 1364–2001) for this project is made available (https://github.com/wonjsohn/stdp_synaptic_competition) for public dissemination.

### Neuron model, synaptic learning model and hardware acceleration

The Izhikevich neuron model is used because it permits the use of biologically realistic variables including transmembrane currents more efficiently in hardware than the more complex Hodgkin-Huxley equations that it approximates (Izhikevich, 2003). In the network, all signals are encoded by neurons as spikes and the spikes pass through synapses. The time resolution of the Izhikevich equation is 1 ms and all of the arithmetic operations in FPGA use a custom-built floating-point arithmetic library which provides fast evaluation time by using combinational logic. This allows minimum clock cycles to update state variables in the on-chip memory, which in turn enables faster-than-real-time emulation of neural activity (Niu et al., 2012). One of the primary reasons to use FPGA technology, in addition to their flexibility and speed, is the inherent parallel computation and the easy scalability. The scalability in number of neurons within a chip is afforded by superimposing parallel sub-network. The total number of neurons emulated in this FPGA is 512 which consists of 128 sub-networks of four neurons circuits representing simple two-layers of neurons described in detail in the subsection 2.2 Neural Structure. Each sub-network is a copy of stochastic versions of the same four neuron circuit, implementing a 1-1 approximation to an all-to-all network. The parallel sub-networks in FPGA are implemented because the statistical behavior of the complete network can be modeled by the average behavior of a large set of identical very small networks with sparse interconnections (Sanger, 2011), enabling approximation of the neural connectivity by sparsely connecting between two neuron pools. The detailed description of the rationale for utilizing the parallel structure in FPGA is described in Niu et al. (2012). It needs to be clarified that in order to focus on the objective of this study, we only show data from randomly selected sub-network of 4-neurons as a sufficient case for demonstration. In this study, the emulation was performed at 190 × real-time, therefore we are able to observe 19 days of neural development within less than three hours. Although the system is designed to easily increase the number of emulated neurons by a 100-fold without sacrificing speed, the current study limits the number of neurons in order to find a sufficient network with minimally simple structure.

### Model of neuron

The Izhikevich neuron is described by the following coupled differential equations with a reset that occurs at the time of an action potential Eqs. (1) and (2):

1 $$\mathrm{v}^{\prime}=0.04\mathrm{v}^{2}+5\mathrm{v}+140-\mathrm{u}+\mathrm{I}$$

2 $$\mathrm{u}^{\prime}=\mathrm{a}(\mathrm{bv}-\mathrm{u})$$

if $\mathrm{v}=30\mathrm{mV},\mathrm{ then v}\leftarrow \mathrm{c},\mathrm{ u}\leftarrow \mathrm{u}+\mathrm{d}$ a, b, c, d: are parameters that describe the spiking behavior of neurons and that can be tuned to emulate different categories of cortical and subcortical neurons. In this implementation, we used typical parameters for regular spiking neurons: a = 0.02, b = 0.2, c = -0.65, d = 8. (Izhikevich, 2003). Other variables include:

*v*: membrane potential.

*u*: membrane recovery variable.

*I*: synaptic currents or injected dc-current.

**Fig. 1:**
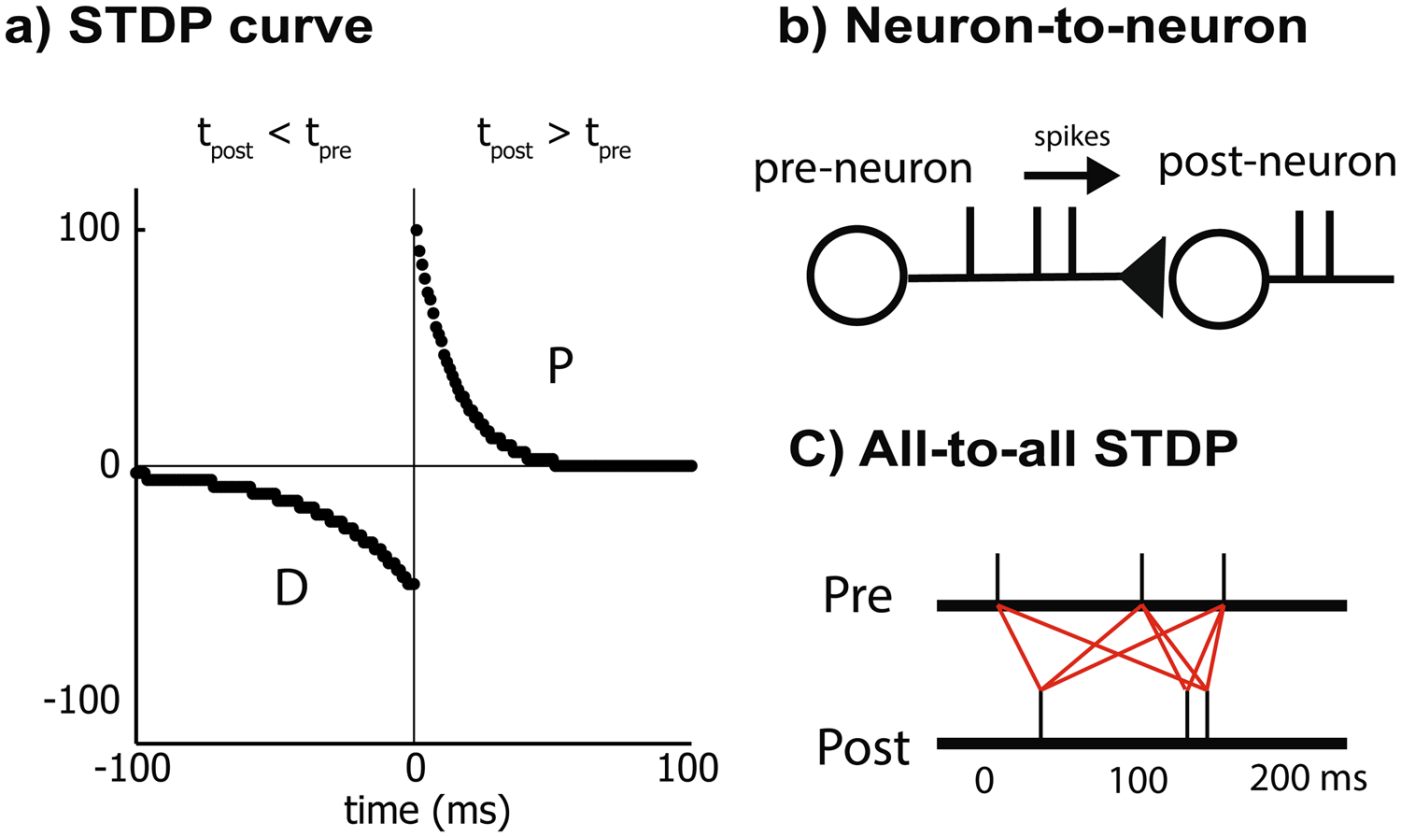
STDP model: the model includes standard all-to-all (Kempter et al., 1999; Song et al., 2000), additive STDP with synaptic decay and stochastic current input to the neuron as an activity generator. **a**) Spike timing dependent plasticity curve implemented on FPGA. Synapse potentiates when postsynaptic spike arrives a few milliseconds after presynaptic spike arrives and depresses if the order is reversed. The parameters A +  = 103%, A- = -51%, tau +  = 0.014 s, tau- = 0.034 s are taken from (Froemke & Dan, 2002). **b**) Illustration of the spikes traveling from presynaptic neurons to postsynaptic neurons via synapses. The postsynaptic current generates postsynaptic spikes. **c**) Illustration of the all-to-all algorithm in STDP. All pairwise combination of presynaptic and postsynaptic spikes contributes to plasticity

The timing of the presynaptic and postsynaptic spikes determines the strengthening and weakening of the connection according to a spike timing dependent plasticity (STDP) model (Bi & Poo, 2001) (Fig. 1). If the presynaptic spike arrives a few milliseconds prior to the postsynaptic spike, then the pair will contribute to long-term potentiation (LTP) of the synapses and vice versa for long-term depression (LTD). The change of the synaptic weight according to the relative timing of pre- and postsynaptic spikes occurs within the STDP kernel or learning window. The window is based on biological responses from studies in rat visual cortex (Froemke & Dan, 2002) where the area under the curve is larger for LTD than for LTP by approximately 1.4 fold. In our implementation, the discretized curve has a time resolution of 1 ms. The standard additive STDP model with all-to-all algorithm (Gerstner et al., 1996; Kempter et al., 1999; Senn et al., 2001; Song et al., 2000) where all pairwise combinations of presynaptic and postsynaptic spikes contribute to plasticity is used (Fig. 1c). In this implementation, maximum time difference between spike pairs is 64 ms due to hardware restrictions. When presynaptic spikes cause postsynaptic spikes to fire, the resulting consistent delay between presynaptic and postsynaptic firing activity facilitates long-term potentiation (LTP), whereas uncorrelated, statistically independent firing activity results in a random relation between the timing of presynaptic and postsynaptic firing which results in long-term depression (LTD).

The time constant for the synaptic weight decay (${\tau }_{SWD})$ is chosen within the reasonable range observed in the biological systems. Here, the time constant of 35 h is used which is within the same order of magnitude of the estimated synaptic strength reduction rate in the cortical synapses (Toyoizumi et al., 2014), and also comparable to the time course between a day (24 h) and a week reported in the transiently increased contrast sensitivity in the visual cortex (Thompson et al., 2008). Because it is a slow decay process, the results of the emulation study are largely insensitive to moderate changes in the value of this time constant. The excitatory postsynaptic current has a time constant of 15 ms (${\tau }_{SCD})$, which is selected within the typical range of a biological system (Roth & van Rossum, 2009).

### Model of synapse

The equations that govern the update of the postsynaptic current and the synaptic weight are as follows Eqs. (3), (4) and (5):

3 $$I\left(n+1\right)=I\left(n\right)+ \delta \left(n-{n}_{spike}\right)g(n)$$

4 $$g\left(n+1\right)=g\left(n\right)+ \sum_{m}\delta \left(n-{n}_{m}\right)\Delta g$$

5 $$\Delta g= f\left({n}_{post}-{n}_{pre}\right)$$

6 $$\tau_{SCD} \frac{dI(t)}{dt} = -I(t)$$

7 $$\tau_{SWD} \frac{dg(t)}{dt} = -g(t)$$

where $${\tau }_{SCD}$$ is the synaptic current decay constant ($$15ms)$$*,* $${\tau }_{SWD}$$ is the synaptic weight decay constant (35 h), *n* is the discrete step that is updated at 1 ms, *n*_*spike*_ is the set of indices of incoming presynaptic spike train, *f* is the STDP kernel in Fig. 1A which is the implementation of the response function, *I* is the postsynaptic current, *δ* is a Dirac delta function, *g* is the synaptic weight, $${n}_{m}$$ is the indices at m^th^ spike occurrence regardless of post and pre, $${n}_{post}-{n}_{pre}$$ is the time difference in discrete representation between pre and post synaptic spike arrival at the m^th^ spike occurrence.

### Neural structure

**Fig. 2:**
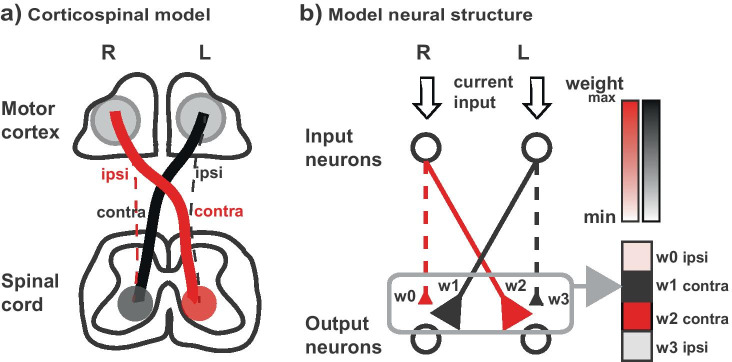
Model neural structure. **a**) Simplified schematic for descending CST. The bold descending lines represent contralateral projection (e.g. right hemisphere to left spinal anterior horn) and the dotted line represents ipsilateral projection. The projection strength of the descending tract is represented by the relative thickness of the lines. In normal development, contralateral projection dominates the ipsilateral projection. **b**) In the simulated neural structure, two layers of neurons representing cortical neurons (input neurons) and output neurons (spinal neurons) are connected via synapses (triangles). A strength of the synapse is represented both by the relative size of the synapses and the thickness of the lines. Red corresponds to the cortical projection from the right hemisphere and black from the left hemisphere

The representation of the descending corticospinal system is modeled in two layers of neurons (Fig. 2). The input neurons in the right and left side (R/L) in the input layer represent the pyramidal neurons at the origin of the corticospinal tract (CST) in the right and left motor cortices. These neurons project to the neurons in the output layer, representing primary motor neurons or interneurons in the spinal anterior horn. Among the four types of synaptic connections present in the structure, the average strength of the connection is represented by the size of the synaptic weight and the thickness of the line connection toward the synapses. Distribution of CST projection within the gray matter is not considered for simplification.

### Simulation procedure

We first demonstrate the three example cases in the activity-dependent synaptic competition. The effect of the initial conditions of the synaptic weights of two input neurons that are under synaptic competition are shown. In these example cases, the input neurons (R/L) receive stochastic input currents that are statistically independent of each other.

Noisy current input to the neuron is used as an activity generator. The pseudorandom values drawn from the standard uniform distribution on the interval ([0.6*${{\varvec{I}}}_{{\varvec{t}}{\varvec{h}}}$, < ${{\varvec{I}}}_{{\varvec{i}}{\varvec{n}}{\varvec{p}}{\varvec{u}}{\varvec{t}}}$< 1.7*${{\varvec{I}}}_{{\varvec{t}}{\varvec{h}}}$], where ${{\varvec{I}}}_{{\varvec{t}}{\varvec{h}}}$ is the neuron firing threshold) flowed into the input neurons which caused stochastic variation in the timing of the spiking events with fixed mean presynaptic firing rate of 15 Hz, which coincides with the average spontaneous firing rate reported in acute brain slice (Bugaysen et al., 2010). The interspike interval of the Izhikevich neuron simulated from the setting had a mean of 67.30 ms, standard deviation of 4.88 ms, and the coefficient of variation of 0.0725 (Fig. S1). The simulation used finite time steps of 1 ms, which is aligned with the time interval of the system, to update the noise value. The current represents constant background stimulation over time. In the context of this study, the goal of the added noise is to create different uncorrelated spike trains from the two input neurons without further consideration to reflect the biophysics of noisy current in the continuous system. The inherent decay rate of the synaptic weight of the contralateral connection is configured to be lower than that of the ipsilateral connection (1:1.5), although the results in the emulation study are largely insensitive to the moderate changes in this ratio as long as the configuration reflects the contralateral connection retaining advantage over the ipsilateral connection in the decay process. This takes into account the factor such as the inherent advantage of the establishment of the contralateral connection over the ipsilateral connection. For example, genetic factors in the development of spinal cord may restrict CST outgrowth from the contralateral to the ipsilateral spinal gray matter and thus promote the establishment of predominantly contralateral termination pattern in the developing CST (Dottori et al., 1998; Kullander et al., 2001; Paixao et al., 2013).

Following the experimental procedure for the feline CP experiment (Martin et al., 2011), starting from the initial conditions of the synaptic weight in the healthy state (contralateral dominance), we varied the input current profile to the neurons in the input layer sequentially as follows:

1. **Unilateral inactivation** which creates an activity-induced bias to bilateral projection from a single hemisphere, with reduced contralateral projection from the inactivated hemisphere.
2. **Bilateral activation** that shows the system’s inability to spontaneously restore the diminished contralateral projection from the inactivated hemisphere, despite restoration of normal bilateral hemispheric activity.
3. **Reverse inactivation** that restores contralateral projection from the previously-inactivated hemisphere by preferential activation of the inactivated hemisphere.
4. **Bilateral activation** that represents normal bilateral inputs without any constraints applied to show that the therapeutic effect persists.

The main outcome measures in the emulation are the time course of the synaptic weights in the simplified two-layer neuronal network in response to the constraints-induced intervention. We intend to focus on identifying *sufficient* conditions that demonstrate the phenomenon of constraint-induced partial reversal of hemiplegia. However, there are no clinically available data that are directly comparable to the time course of the synaptic strength; we are only aware of the fact that typically CIMT requires 2–4 weeks of training, 4–6 h per day (Brady & Garcia, 2009; Taub et al., 1999, 2007). For this reason, the comparison from the emulation is focused on demonstrating the characteristics of the “four stages” in a qualitative way. These stages in emulation are posed to parallel both the development of and the recovery from the hemiplegic CP in Martin’s experiment (Friel & Martin, 2007). The duration of each stage is configured to last sufficiently long to observe the convergence of synaptic weights toward either extreme (max or minimum weight) except in the reverse inactivation stage. The reverse inactivation stage is designed to end at the point when the weak contralateral synaptic connection was potentiated more than its ipsilateral competitor with an acknowledgement that in a real brain with a unilateral stoke, the injured hemisphere may not be strengthened to the extent that it would dominate the uninjured hemisphere.

## Results

### Demonstration of plasticity effect with STDP

**Fig. 3:**
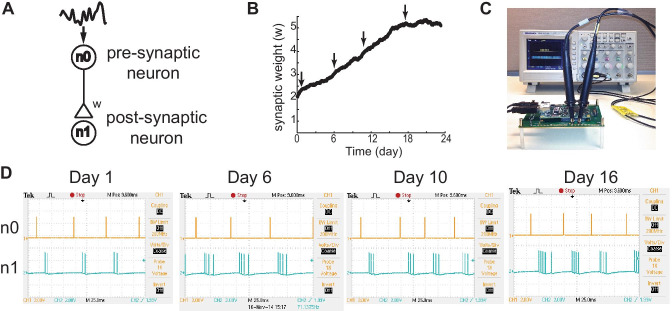
Validation of STDP implementation using neuromorphic hardware. The monosynaptic connection between two neurons are continuously activated by pseudo-random input (**a**). The neuromorphic hardware enabled emulating the effect equivalent of 23 days of continuous activation in 2 h and 55 min due to the 190 × real-time acceleration. The time course of the gradual change in the synaptic weight is shown in (**b**). At various time points, an effect of a pre-synaptic spike to a post-synaptic firing are shown in the snapshots from an oscilloscope (**c** and **d**). A gradual increase in post-synaptic burst size in response to each pre-synaptic spike is observed as the synaptic weight increases over time.

Figure 3 demonstrates the functionality of STDP implemented in the neuromorphic hardware. When the emulation is set up to connect two neurons (Fig. 3a), the pre-synaptic neuron is continuously activated by an input current composed of constant current superimposed with noise. The current produces a firing rate of about 15 Hz in the pre-synaptic neuron (n0). The time course of the synaptic weight (w) in response to the 23 days of continuous activation is shown in Fig. 3b. Note that for this specific recording the synaptic weight appears to have reached a plateau after the 18^th^ day before reaching any hard upper bound because a heightened synaptic decay rate was used. For the rest of the results, the synaptic decay rate ( ${\tau }_{SWD}$) indicated in the methods was used. Since the change in synaptic weight is solely determined by the occurrence of the LTP and LTD, this validates that our emulation is capable of utilizing the characteristics of the STDP-based updates in the synaptic weights. When the nodes for the pre- and post-synaptic neurons are probed with an oscilloscope (Fig. 3c), it is verified that initially a presynaptic spike elicits ~ 2 spikes in the post-synaptic neuron n1 Fig. 3d). After 23 days of continuous activity, which corresponds to ~ 2.9 h of emulation time due to 190 × real-time acceleration, increased number of post-synaptic spikes are observed upon the same single pre-synaptic spike input. The corresponding synaptic changes are the accumulated effect of 29.8 million presynaptic spikes involving the 23 days period.

### Example cases for activity-dependent synaptic competition

**Fig. 4:**
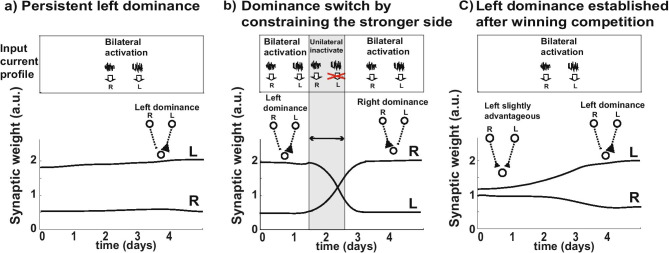
Three example cases for synaptic competition according to STDP when two input neurons project to an output neuron. The change in synaptic weights representing the connection strengths between input and output neurons are plotted with different initial conditions. The STDP model dictates that the relationship between pre- and postsynaptic spikes will induce long-term potentiation (LTP) of the synapse if input and output are correlated, and long-term depression (LTD) if input and output are not correlated. In all three example cases, the input neurons (R/L) receive stochastic input currents that are statistically independent from each other. **a**) When the simulation starts with a left predominant initial condition, the predominant connection suppresses the growth of the weaker connection, thus the states are stuck at left dominance. **b**) Transient constraint of input activity in the dominant side induces a switch in the predominant connection. **c**) When the left is considerably stronger than the right, the synaptic competition will establish the left dominance.

Figure 4 demonstrates the effect of the initial conditions of the synaptic weights of two input neurons that are under activity-dependent synaptic competition. When the two input neurons project to an output neuron to compete for establishing their connection, the initial conditions of the synaptic weights determine the outcome of the competition—the predominant initial connection is likely be the winner that “takes all” (Fig. 4a-c). This is due to the rule of STDP which ensures the suppression due to the LTD in the weaker connection outweighs the LTP when the two competing connections receive statistically independent input signals, e.g. uncorrelated pre-postsynaptic activity leads to LTD. However, the winner can be switched with an intervention. A transient constraint of input activity in the dominant side induces a switch in the predominant connection (Fig. 4b). In other words, the transient blockade of the activity, an absence of any activity, to the dominant projection provides inhibition of the suppression which previously prevented the disadvantaged connection from increasing.

Activity-dependent synaptic competition as a key mechanism for recovery from a damaged corticospinal tract following constraint-induced intervention

**Fig. 5:**
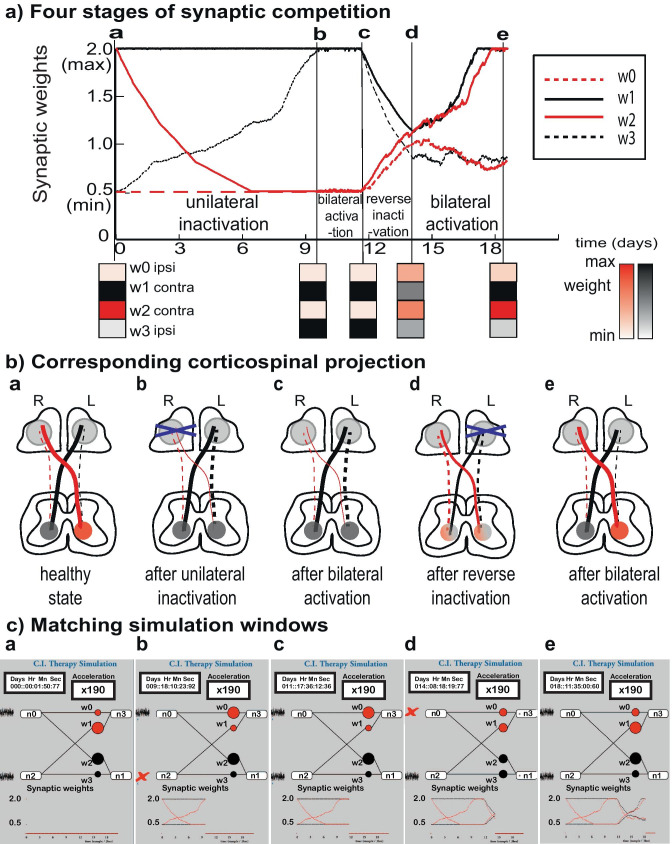
Simulating constraint-induced therapy by activity-dependent STDP. **a**)** Change of synaptic weight.** Change of synaptic weights of 4 example synapses according to the four stages of input current profiles. The 4 × 1 weight matrix helps to visualize the relative strength of the four synaptic connections. Note that w0 and w1 are in competition and likewise w2 and w3 are in competition**. b**)** Schematics of corresponding corticospinal projections.** a. The healthy state with predominant contralateral projections. b. After unilateral inactivation (blocking R). c. After bilateral activation d. After reverse inactivation (blocking L). e. After bilateral activation. **c**)** Matching simulation windows.** Snapshots of the matching simulation windows at each point corresponding to B. Note that the direction of the signals in this window is drawn from left to right. The miniature plot in the bottom keeps track of the real-time change of the synaptic weights. The magnified version is in A. The results shown are from a typical 4-neuron subnetwork selected from the neuron pool

A schematic of CST in the four stages according to the input current characteristics (Fig. 5b) and the corresponding snapshots of simulation windows (Fig. 5c) are shown in Fig. 5. The simulation window displays the four stages parallel both the development of and the recovery from the hemiplegic CP in Martin’s experiment (Friel & Martin, 2007). Simulated windows for the four stages corresponds to the compartments in Fig. 5a. The results shown are from a randomly drawn 4-neuron subnetwork selected from the output neuron pool.

1. **Initial healthy stable state**
  Initial stable state has normal predominant contralateral projection and weaker ipsilateral projection.
2. **Unilateral inactivation** creates hemiplegic bilateral projection
  Unilateral inactivation (blocking the input current to R) causes the ipsilateral projection from the active hemisphere (w3) to grow because the suppression from the activity in the dominant projection is removed, resulting in the development of bilateral projection from the same hemisphere (w1, w3). The contralateral projection from the blocked hemisphere weakens (w2) due to inactivity and synaptic decay.
3. **Bilateral activation** shows the system’s inability to spontaneously restore the diminished contralateral projection
  Once the hemiplegic bilateral projection is established, it is demonstrated that resuming the bilateral activation in both hemispheres does not spontaneously restore the diminished contralateral projection (w2) back to the initial healthy state. In other words, the state is *stuck* in a hemiplegic state (as Fig. 4a). This is due to the fact that suppression from the strong ipsilateral projection (w3) stifles the growth of contralateral projection from the opposite hemisphere (w2).
4. **Reverse inactivation** enables the diminished contralateral projection to be restored
  Reverse inactivation (blocking the input current to L) enables the diminished contralateral projection (w2) to be restored by transiently removing the suppression from the now-dominant ipsilateral projection (w3) (as in Fig. 4b). The duration of this transient phase is an experimental design choice. The duration should be prolonged until the effect of the constraint leads to contralateral projection being slightly stronger than the pathologically developed ipsilateral projection (as in Fig. 4c), i.e., w1 > w0, w2 > w3.
5. **Bilateral activation** that represents normal bilateral inputs without any constraints applied to show that the therapeutic effect is stable and persists
  Bilateral activation after the reverse inactivation resumes the competition between contralateral and ipsilateral projection for the synaptic connection to the output neurons. If the reverse inactivation stage ended at contralateral connection being considerably stronger than the ipsilateral connection, the competition leads to the contralateral dominant state.

## Discussion

Constraint-induced movement therapy (CIMT) is effective in producing improvement in limb use of patients after cerebrovascular accidents (CVA) (Miltner et al., 1999; Taub et al., 1999). It was previously hypothesized as a “synaptic competition rule” in which the more active/used connections are more competitive and overtake less active/used connections (Friel et al., 2014). Here, we have provided an emulation of synaptic competition based on known properties of spike-timing dependent plasticity. In the 19-days of continuous emulation which includes periods of the development of hemiplegia and the recovery from it due to imposing an alternate constraint on the uninjured hemisphere, we observed that activity-dependent synaptic competition is a sufficient mechanism to account for persistent deficits that may improve following CIMT.

In our demonstration, **unilateral inactivation** created a “hemiplegic” bilateral projection because the silenced hemisphere caused the ipsilateral projection from the active hemisphere to grow according to activity-dependent STDP. The injured side does not have capacity to maintain connection and the subsequent growth of synapses from the uninjured side further suppresses the regrowth of the injured side and leads to persistence of the deficit—the vicious circle model (Martin, 2012). Plasticity causes the system to be stuck at a hemiplegic state indefinitely, even with the normal bilateral activation profile, until **reverse inactivation** is applied as a constraint-induced intervention. Reverse inactivation enables the diminished contralateral projection to be restored by transiently removing the suppression from the now-dominant ipsilateral projection.

### Parallel theory in the visual system: amblyopia

Amblyopia, a common visual cortex disorder caused primarily by binocular disruption during an early critical period, is often considered to be the sensory equivalent of hemiplegic CP (Eyre et al., 2007) because of the similarity in the competitive sensory activity-dependent refinement process of binocular vision. The disorder is usually diagnosed as reduced visual acuity in the otherwise healthy eye (Holmes & Clarke, 2006). Primary visual cortex responds to binocular input, and the unmatched images seen by the two eyes in early life results in vision in one eye being suppressed (Jampolsky, 1955; Joosse et al., 1999; Pratt-Johnson, 1984; Travers, 1938), by a process of activity-dependent plasticity. Evidence from previous studies support an idea that individuals with amblyopia have a capacity for binocular vision, but this capacity is suppressed or inhibited under normal viewing conditions (Hess et al., 2014). Recently developed quantitative measures for suppression such as the motion coherence test, the orientation coherence test, the phase test support that a stronger suppression is associated with a greater acuity deficit (Li et al., 2011) which is since corroborated by other studies (Li et al., 2013; Narasimhan et al., 2012; Zhou et al., 2013). The shared interpretation from these studies is that the amblyopic signal is weaker, noisier, and maybe strongly suppressed by signals in the fellow eye. This suppression, therefore, within the context of binocular vision, is considered a primary target for amblyopia treatment because its elimination is a necessary step in any binocular restoration (Hess et al., 2010, 2014).

**Fig. 6:**
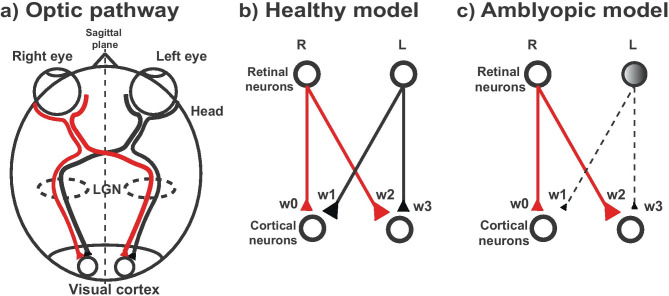
Simplified amblyopia model of the visual system. **a**) Schematic of a simplified optic pathway. Information from each eye projects to visual cortex for both hemispheres. **b**) Model for a healthy visual system. **c**) Hypothesized model for an amblyopic visual system. Monocular deprivation in the left eye leading to reduced visual function in the left eye, perpetuated by the suppression from the fellow eye

Traditional treatment for amblyopia consists of forcing the use of the amblyopic eye by patching the fellow eye. The rationale behind patching is that depriving the sound eye of vision removes suppression of vision in the amblyopic eye and allows the visual experience to promote recovery of monocular functions such as visual acuity in that eye and eccentricity of fixation (Holmes et al., 2003). Parallel to the CST model in this study, the structure of the competitive interaction between afferent pathways from binocular pathway can be represented as the two-layer neural model in this study—retinal neurons as an input layer and the cortical neuron in the binocular zone of the visual cortex as an output layer (Fig. 6). Although structural and functional plasticity in primary visual cortex during the process of ocular dominance plasticity (ODP) involves multiple layers and functional architectures (e.g. lateral geniculate nucleus, pars dorsalis LGNd, layer 4 and layer 2/3) (Adams & Horton, 2009; Espinosa & Stryker, 2012), with the reduction of relays while focusing on the binocular competition, the dual-layer is capable of demonstrating the ocular competition at the binocular zone in the primary visual cortex. Under this assumption, the genesis of amblyopia by monocular deprivation (MD) corresponds approximately to the unilateral inactivation in the hemiplegic CP model, and the patching or suturing of the lids of the sound eye corresponds to the reverse inactivation phase.

Although the model is capable of demonstrating the principle of constraint-induced intervention that targets the suppression mechanism in the binocular visual system, the model predicts only limited phenomena in amblyopia. For example, more recently-discovered short-term binocular plasticity due to a *lack* of sensory stimulation cannot be explained in the same level of abstraction afforded by this model (Zhou et al., 2013). The re-balancing of the binocular functions reported in the inverse occlusion (occlusion of the amblyopic eye) requires additional complexity of modeling. The fact that the monocular effect taking more time to develop than binocular effects and the former experiment focuses exclusively on the monocular function such as visual acuity and eccentricity of fixation suggests that the two effects could have different underlying mechanisms that are distinct from each other (Zhou et al., 2013). The fact that the traditional treatment approach of patching the fellow sighted eye will be strengthened, rather than replaced, by the inverse occlusion treatment also suggests the two types of treatments are in supplementary relationship rather than in a contradictory relationship.

### BCM and spike-based plasticity model

In the “BCM” neuron model, ongoing adjustment of the threshold for potentiating or depressing Hebbian synaptic strength is termed “homeostatic plasticity”. Changes in the threshold due to monocular deprivation are hypothesized to be the mechanism for persistence of monocular dominance in amblyopia. The BCM equations are based on average firing rates rather than individual spikes, and differences in threshold for different inputs provides a mechanism by which one class of inputs may be continually depressed despite some degree of input–output correlation (Bienenstock et al., 1982; Cooper & Bear, 2012). Although certain forms of STDP can be manipulated by adjusting the parameters of the learning rule, shape of the STDP kernel, etc., to capture some of the features of BCM (Izhikevich & Desai, 2003; Pfister & Gerstner, 2006), the two classes of learning models remain a competing phenomenological model of synaptic plasticity in a biological system.

**Fig. 7:**
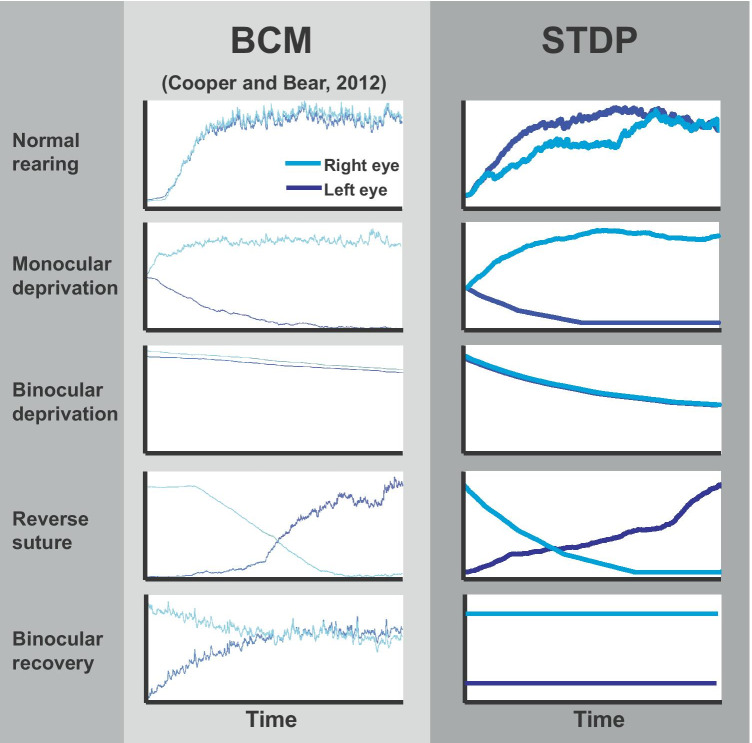
BCM and STDP in qualitative agreement in simulating monocular deprivation and the effect of reverse suturing the sound eye. (BCM column data from (Cooper & Bear, 2012), reprinted with permission.) Left column: BCM simulation of the inputs from the two retinas onto a single cortical neuron. Right column: STDP simulation of the inputs from two retinas on to a single cortical neuron in visual cortex. From top to bottom, normal rearing, monocular deprivation, binocular deprivation, reverse suture (constraint) and binocular recovery are demonstrated. Time scale is arbitrary, as in (Cooper & Bear, 2012)

Figure 7 compares the predictions of the STDP and BCM models for the development and treatment of amblyopia due to monocular deprivation. The left column shows a BCM simulation of the inputs from the two retinas onto a single cortical neuron under five conditions: normal rearing, monocular deprivation, binocular deprivation, reverse suture, and binocular recovery(Cooper & Bear, 2012). Right column shows a prediction of our STDP model of the inputs from two retinas on to a single cortical neuron in a visual cortex for the like conditions from the left column, using the Izhikevich neurons and STDP model described in the methods. Note that the condition for the monocular deprivation is identical to that of the *unilateral inactivation* in Fig. 5, the reverse suture to the *reverse inactivation*, and the binocular recovery to the *bilateral activation*. STDP is able to produce qualitatively similar outcome for the first four conditions. Interestingly, only STDP shows persistence of the deficit, whereas BCM trends back toward binocular vision over time (“binocular recovery” in Fig. 7). This would be an important difference and if so, may invalidate BCM as a model for persistent hemiplegia following unilateral cortical inactivation or deprivation.

### Clinical implication

In this study, it is demonstrated that the persistence of suboptimal stable solutions can be due to an unwanted hysteresis effect of STDP: once the input pattern allows unilateral dominance to be established, decreased activation of the disadvantaged side will persist. STDP is the synaptic mechanism that allows the persistent stable suboptimal solution. Given that this mechanism may be widely prevalent in the CNS, the same principle could be operating wherever multiple input neurons compete for an output neuron—whether it is at the spinal or cortical level. Our results do not address nor explain the presence of an apparent “critical period”. For hemiplegic CP, it is vitally important that a treatment will be most effective if applied early in development. In Amblyopia, the patching treatment is known to work best when the occlusion is initiated before 3 years of age (Epelbaum et al., 1993), although some recent studies reported that adult human visual cortex has a significant degree of plasticity (Sasaki et al., 2010). It is certainly possible that in the context of years of abnormal function, additional plasticity or developmental mechanisms supervene that lead to irreversible asymmetric innervation.

### Synapse model sensitivity

Although it is not our interest in this study to test the different effects of various implementations of STDP mechanisms, it can be said that the result demonstrated in this study is generalizable if (1) update mechanisms dictate that correlated pre- and postsynaptic spikes cause potentiation and uncorrelated pre- and postsynaptic spikes cause depression of the synaptic weight, (2) postsynaptic firing rates are not in the range that results in net depression. Although the time constant of synaptic current and weight decay is not sensitive to the qualitative results for this study directly, the time constant for synaptic weight decay (${\tau }_{SWD}$) requires to be much bigger (e.g. by several order of magnitude) than that of the synaptic current. Our implementation has an asymmetric STDP curve, only excitatory synapses, and is all-to-all and additive. Variation in curve shape, nearest-neighbor implementation instead of all-to-all, or perhaps multiplicative instead of additive updates will likely still generate the same phenomenon, but this remains to be tested experimentally.

## Conclusions

While our neuromorphic emulation with limited biorealism does not prove the biological mechanism, it provides a conceptual model and demonstrates that even such a very simple model is sufficient to explain the phenomenon without inclusion of more sophisticated neuronal connections. The result occurred due to STDP alone, without the introduction of hypothesized homeostatic thresholds as used in BCM-type models. Therefore, persistence of plasticity-mediated phenomena does not provide strong support for the existence of BCM-type homeostatic plasticity. The minimal requirements of our emulation suggest that it is likely that any system with STDP and competing inputs may be susceptible to this type of plasticity-mediated persistent deficit, and may be amenable to constraint-like treatment.

## Supplementary Information

Below is the link to the electronic supplementary material.

Supplementary file1 (DOCX 33 KB)
