# Supplementary material for: Constraint-induced intervention as an emergent phenomenon from synaptic competition in biological systems
Source: J Comput Neurosci. 2021 Apr 6;49(2):175–88. doi: 10.1007/s10827-021-00782-9 (PMC8046695; doi:10.1007/s10827-021-00782-9)
Supplement: Supplementary file 1 — Supplementary file1 (DOCX 33 KB) [file 10827_2021_782_MOESM1_ESM.docx]

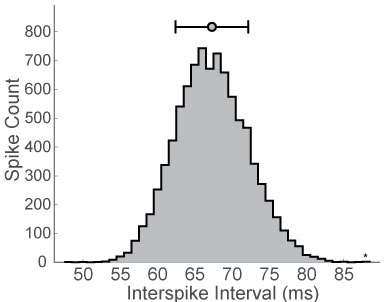


**Figure S1**. Interspike interval recorded from the 8,914 spikes from the Izhikevich neuron had a mean of 67.30ms, a standard deviation of 4.88ms, and the coefficient of variation of 0.0725, which was simulated in MATLAB under the same input characteristic to the neuron model in the input layer.
